# Potentiation of excitatory transmission in substantia gelatinosa neurons of rat spinal cord by inhibition of estrogen receptor alpha

**DOI:** 10.1186/1744-8069-6-92

**Published:** 2010-12-11

**Authors:** Yan-Qing Zhong, Kai-Cheng Li, Xu Zhang

**Affiliations:** 1 Institute of Neuroscience and State Key Laboratory of Neuroscience, Shanghai Institutes for Biological Sciences, Chinese Academy of Sciences; 2 Department of Diving Medicine, the Naval Medical Research Institute, Shanghai, China

## Abstract

**Background:**

It has been shown that estrogen is synthesized in the spinal dorsal horn and plays a role in modulating pain transmission. One of the estrogen receptor (ER) subtypes, estrogen receptor alpha (ERα), is expressed in the spinal laminae I-V, including substantia gelatinosa (SG, lamina II). However, it is unclear how ERs are involved in the modulation of nociceptive transmission.

**Results:**

In the present study, a selective ERα antagonist, methyl-piperidino-pyrazole (MPP), was used to test the potential functional roles of spinal ERα in the nociceptive transmission. Using the whole-cell patch-clamp technique, we examined the effects of MPP on SG neurons in the dorsal root-attached spinal cord slice prepared from adult rats. We found that MPP increased glutamatergic excitatory postsynaptic currents (EPSCs) evoked by the stimulation of either Aδ- or C-afferent fibers. Further studies showed that MPP treatment dose-dependently increased spontaneous EPSCs frequency in SG neurons, while not affecting the amplitude. In addition, the PKC was involved in the MPP-induced enhancement of synaptic transmission.

**Conclusions:**

These results suggest that the selective ERα antagonist MPP pre-synaptically facilitates the excitatory synaptic transmission to SG neurons. The nociceptive transmission evoked by Aδ- and C-fiber stimulation could be potentiated by blocking ERα in the spinal neurons. Thus, the spinal estrogen may negatively regulate the nociceptive transmission through the activation of ERα.

## Findings

Several studies suggest that estrogen plays an important role in the spectrum of neural functions, such as nociception [1-4]. Estrogen is synthesized in many neurons in laminae I-III of the spinal cord [5-8], and potentiates the pain behavior [8]. Estrogen may modulate nociceptive responses through the increase of glutamate-induced currents, the inhibition of γ-aminobutyric acid (GABA) and glycine (Gly) receptors, or the modulation of the opioid receptors in the spinal dorsal horn [9-11]. It is well known that the classical estrogen action in neurons is to activate nuclear estrogen receptor α and β (ERα/β), which cause long-term genomic effects [12,13], or to activate cytoplasmic signaling events at or near the plasma membrane [14,15] through either membrane-localized classical ERs [16,17] or novel ERs [18]. Recent studies showed that ERα is expressed in spinal laminae I-V, especially in laminae I-II, and is most abundant in the lower lumbar (L) and sacral segments [19,20]. However, whether the ERα is involved in estrogen-mediating pain behavior remains unclear. Considering that the superficial dorsal horn of the spinal cord, especially substantia gelatinosa (SG, lamina II), plays an important role in the modulation of synaptic transmission of fine myelinated A (Aδ)- and unmyelinated C-afferent fibers [21,22], we used a selective ERα antagonist, methyl-piperidino-pyrazole (MPP) [23], to examine the function of spinal ERα in nociceptive transmission in SG neurons. The dorsal root-attached spinal cord slices were prepared from adult rats and recorded with whole-cell patch-clamp technique.

**Figure 1:**
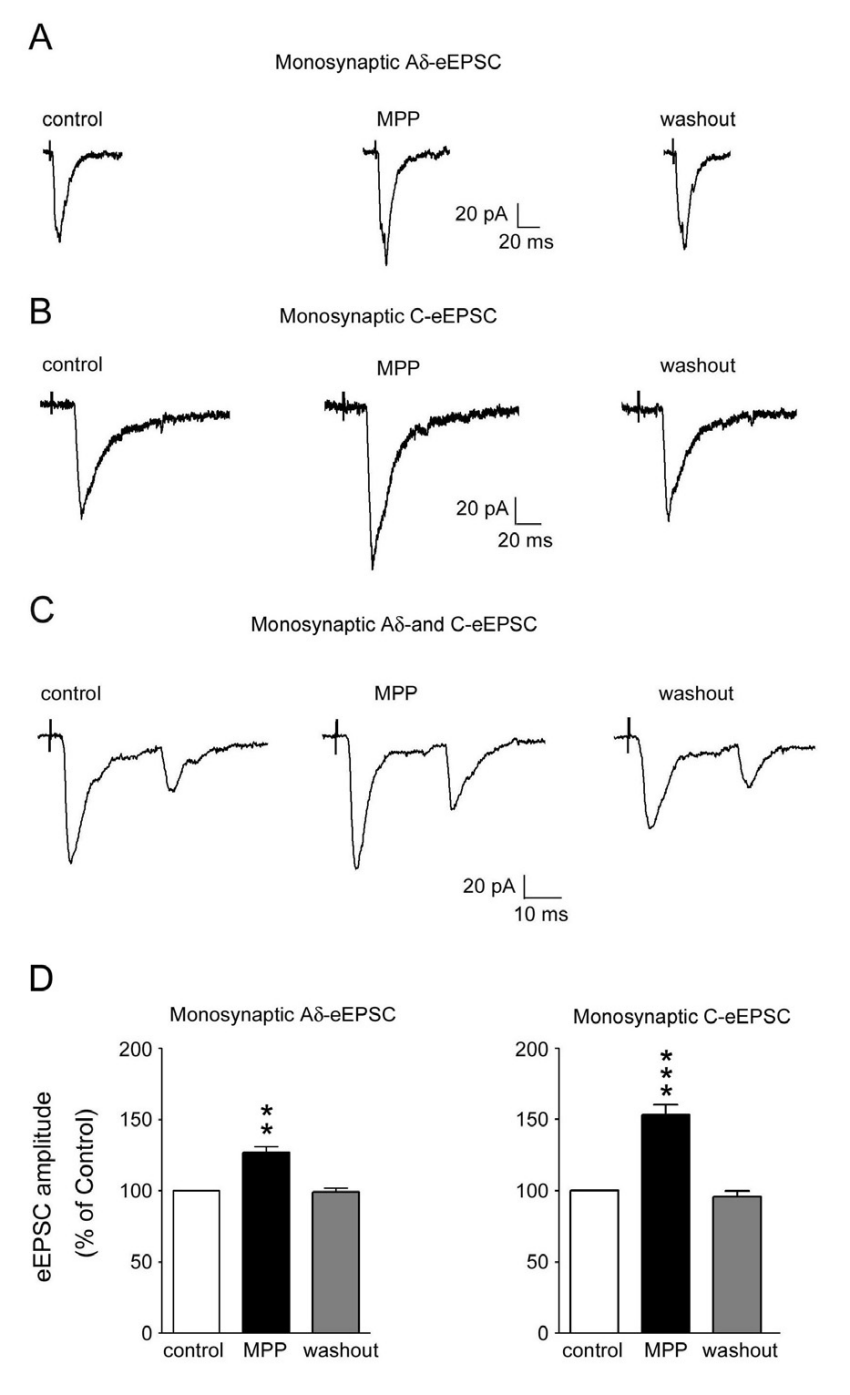
**Effects of MPP on monosynaptic Aδ- or C-fiber eEPSCs in SG neurons**. (A) Average traces of six consecutive Aδ-eEPSCs (stimulated at 0.2 Hz) before (left), during the treatment with MPP (10 μM, middle), and 5 min after washout (right) are shown. (B) Average traces of six consecutive C-eEPSCs (stimulated at 0.2 Hz) before (left), during the MPP treatment (10 μM, middle), and 5 min after washout (right) are shown. (C) Effects of MPP (10 μM) on monosynaptic Aδ- and C-fiber eEPSCs were obtained from a single neuron. Average traces of six consecutive eEPSCs (stimulated at 0.2 Hz) before (left), during the MPP treatment (middle), and 5 min after washout (right) are shown. (D) The MPP-induced increase in the amplitude of C-eEPSCs is more pronounced than that of Aδ-eEPSCs in SG neurons. Peak amplitudes of monosynaptic C-eEPSC and Aδ- fiber eEPSCs in the presence of MPP (10 μM) (n = 5 and 8 neurons, respectively) were analyzed. ** *P *< 0.01, *** *P *< 0.001 (paired t-test).

Whole-cell recordings were carried out in SG neurons. Stable recordings could be maintained in vitro for more than 8 hrs; and recordings could be made from a single SG neuron up to 2 hrs. The monosynaptic, Aδ-afferent evoked excitatory postsynaptic currents (eEPSCs) with a mean amplitude of 156 ± 25 pA (50~360 pA; V_H _= -70 mV) were found in ~70% of recorded neurons (18/25). In 8 out of these 18 neurons (~ 45%), superfusion of MPP (10 µM) increased the peak amplitude of the Aδ-eEPSC in a reversible manner (Figure 1A). The enhancement was averaged at 130 ± 5% (n = 8) in magnitude.

The monosynaptic C-afferent eEPSCs with a mean amplitude of 135 ± 31 pA (40~310 pA; V_H _= -70 mV) were found in ~60% of neurons (10/16). In 5 out of these 10 neurons, MPP (10 μM) treatment increased the peak amplitude of the C-eEPSC and normal Kreb's solution washed off the MPP-induced effect (Figure 1B). The averaged magnitude of the enhancement was 150 ± 6% (n = 5). In other three neurons exhibiting both Aδ- and C-eEPSCs, MPP increased the amplitude of both types of eEPSCs (Figure 1C).

Further comparison of MPP-induced enhancement between Aδ- and C-eEPSCs showed that the increase in C-eEPSC amplitude during MPP application was more pronounced than that of Aδ-EPSC (Figure 1D). In spite of the differences of their sensitivity to MPP, Aδ- and C-eEPSCs were responded with a similar time course following MPP superfusion. The current amplitudes had been changed maximally and measured at 3 min after MPP was applied.

**Figure 2:**
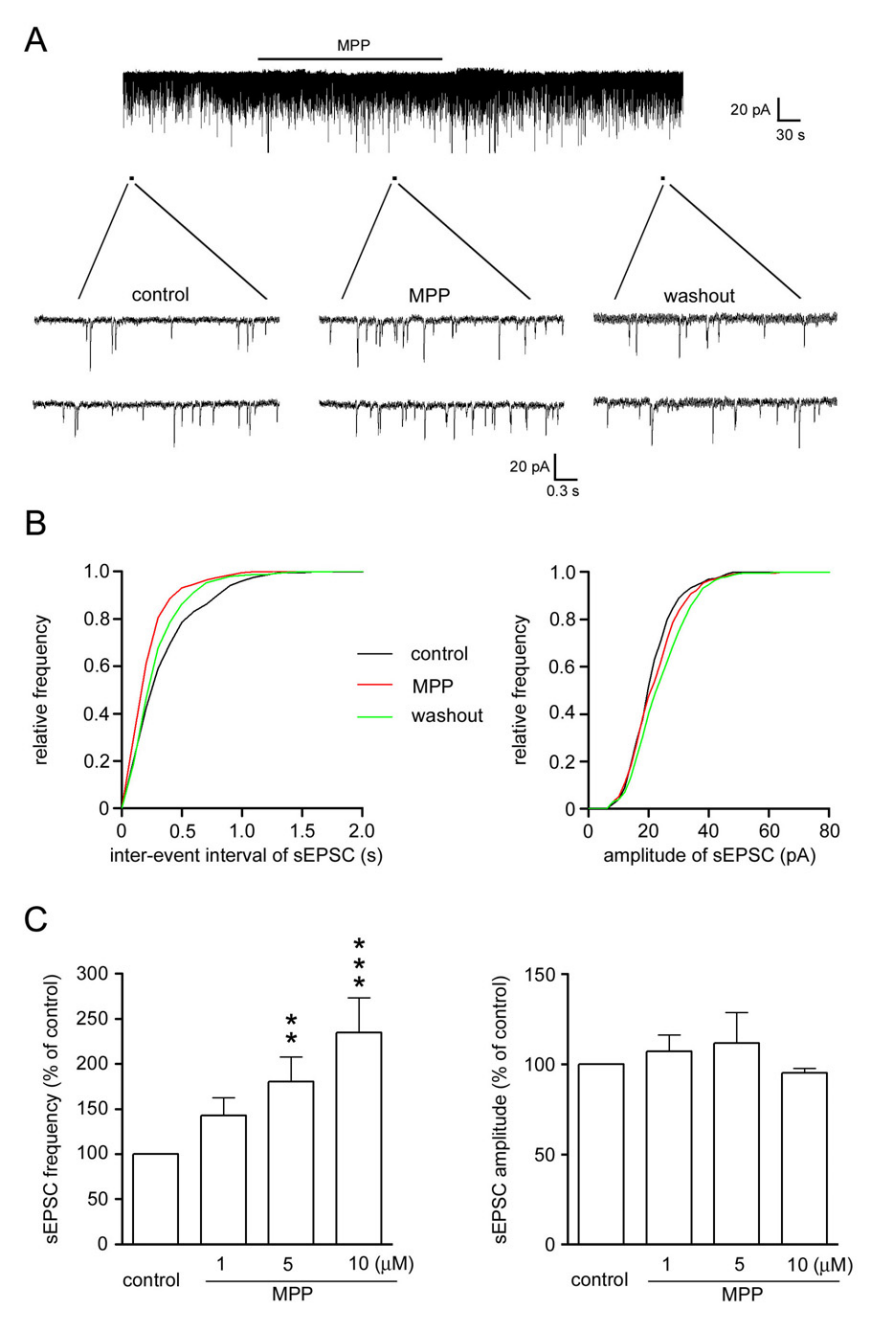
**MPP-induced increase in sEPSC frequency**. (A) Traces of sEPSCs before (left) and during the MPP treatment (10 μM) (middle; 3 min after the beginning of MPP application), and after washout (right) are shown. (B) Cumulative distributions of the inter-event interval (left) and amplitude (right) of sEPSC before (black line), during the MPP treatment (red line) and washout (blue line) (n = 393, 339 and 321 sEPSC events over 1 min, respectively) are analyzed. (C) MPP only enhanced the frequency of sEPSC, but not their amplitude, in a dose-dependent manner. ** *P *< 0.01, *** *P *< 0.001 (paired t-test).

To examine whether MPP modulated the afferent synaptic transmission through pre- or post-synaptic action, the spontaneous EPSC (sEPSC) in SG neurons during MPP treatment were analyzed. We found that superfusion of MPP (10 μM) resulted in a reversible enhancement in sEPSC frequency (Figure 2A and 2B; 234 ± 9% of control at 3 min following its application, n = 8; P < 0.001). Furthermore, the MPP-induced responses were dose-dependent. At a concentration of 1 and 5 μM, the increased sEPSC frequency was 124 ± 8% (n = 5) and 180 ± 6% (n = 5), respectively. However, the amplitude of sEPSC was not altered by the treatment with MPP (Figure 2B and 2C). The modulation on sEPSC frequency, but not on amplitude, suggests that MPP regulates nociceptive transmission through a pre-synaptic action.

**Figure 3:**
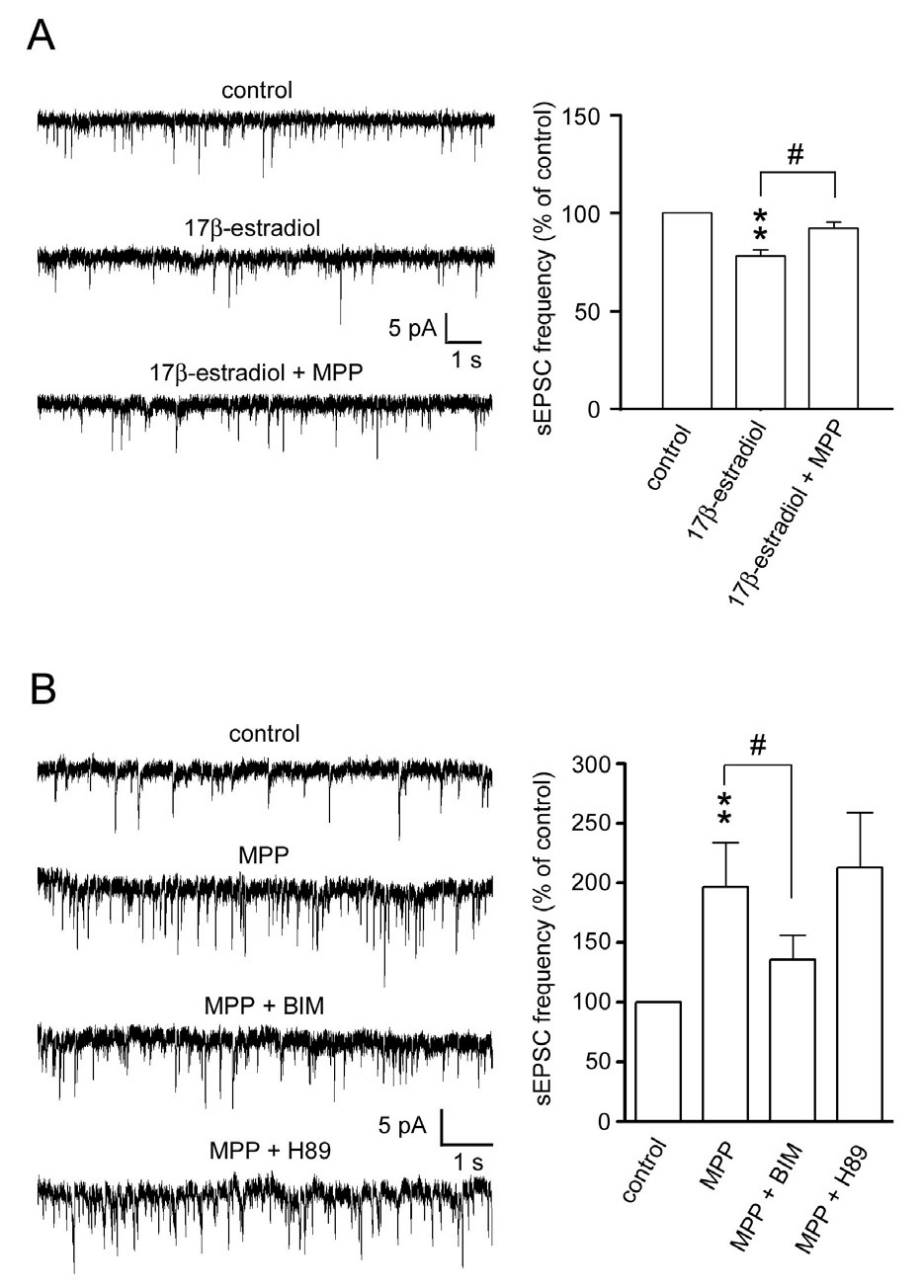
**Exogenous estrogen-induced reduction of sEPSC frequency and the involvement of PKC in MPP-induced increase of sEPSC frequency**. (A) Traces of sEPSCs before the treatment (control), during the treatment with 17β-estradiol (1 μM), and during the treatment with 17β-estradiol (1 μM) plus MPP (10 μM) are shown. Quantitative analysis shows that estrogen reduces sEPSC frequency, and MPP reversed the estrogen-induced inhibition (n = 6). (B) Traces of sEPSCs before the treatment (control), during the MPP treatment (10 μM), during the treatment with MPP (10 μM) plus BIM (1 μM), and during the treatment with MPP (10 μM) plus H89 (5 μM) are shown. Quantitative analysis shows that BIM, but not H89, inhibits MPP-induced increase in sEPSC frequency (n = 5). ** *P *< 0.01, ^# ^*P *< 0.05 (paired t-test).

To investigate whether exogenous estrogen could modulate glutamatergic excitatory synaptic transmission in SG neurons, we tested whether a treatment with 17β-estradiol could regulate the sEPSC. We found that the frequency of sEPSC was reduced by bath-applied 17β-estradiol (1 μM), and this effect could be reversed by MPP (Figure 3A, n = 6). Finally, we also found that a PKC inhibitor bisindolylmaleimide I hydrochloride (BIM) (1 μM), but not a PKA inhibitor H89 (5 μM), could reduce the MPP-induced enhancement of sEPSC frequency (Figure 3B, n = 5).

The above results suggest that nociceptive transmission could be facilitated by blocking ERα, such as a selective antagonist MPP used in the current study. In addition, the endogenous estrogen may activate ERα in spinal dorsal horn to reduce glutamatergic excitatory transmission and inhibit the nociceptive responses. Previous studies showed that ERα is expressed in the small-diameter neurons in the dorsal root ganglion (DRG), a subset of nociceptive sensory neurons [24-26]. The ERα-mediated inhibition of ATP-induced Ca^2+ ^signaling in mouse DRG neurons [27] suggests that peripheral ERα negatively regulates nociceptive transmission. Moreover, ERα immunoreactivity has been found in the spinal cord. A larger numbers of ERα-immunoreactive neurons were found in the lower lumbar spinal cord segments. These ERα-containing neurons were mainly found in the spinal lamina II, and some were in laminae I, III, IV, V, and X. In the superficial layers of the medullary dorsal horn, ERα-immunoreactivity was mainly located in lamina II, which was also expressed noxious-induced Fos [19,20,28]. These findings provide an anatomical and neurochemical basis for the hypothesis that estrogen activates ERα directly to regulate pain transmission at the central level [28]. Consistent with early studies, our present study shows that in the spinal dorsal horn, ERα is involved in the modulation of nociceptive Aδ- and C-afferent transmission.

Previous studies showed that the enzyme aromatase catalyzed the formation of estrogen from testosterone in the gonads and other tissues, such as many nociceptive neurons in the spinal laminae I-III. Moreover, the specific nonsteroidal aromatase inhibitor, vorozole, was found to inhibit the phosphorylation of aromatase in the spinal cord and induce an acute inhibition of the endogenous spinal estrogen synthesis, which could consequently lead to the inhibition of nociceptive responses [6-8]. Some studies showed that estrogen rapidly potentiated glutamate (kainate)-induced currents through a second-messenger cascade [9]. G-protein coupled to inwardly rectifying K channels could be inhibited by the estrogen-induced reduction of the potency of GABA and opioid receptor agonists [18,29]. Moreover, estrogen inhibited the GABA and Gly receptors or modulated the opioid receptor in the spinal dorsal horn [10,11]. The estrogen-induced potentiation of kainate currents and inhibition of GABA and Gly receptors may play a role in the activation of the central pain pathways [30-32]. Our present results show that activation of ERα by estrogen may inhibit nociceptive transmission through a PKC signaling pathway. Therefore, the ERα-mediated negative regulation of nociceptive transmission may be balanced by the effect of estrogen on other receptors in some certain extent.

In conclusion, we propose that estrogen may inhibit the nociceptive transmission via the ERα in the spinal dorsal horn. Our results may help to understand the functions and mechanisms of estrogen in pain modulation, and suggest that ERα may be a potential target in relieving pain syndrome.

## Materials and methods

### Spinal slice preparation and whole-cell recording

Transverse spinal cord slices (~600 μm, L4 or L5 segment) with an attached dorsal root from adult rats (male, 6-8 weeks old) were prepared with a vibrating microslicer and perfused in the oxygen-bubbled Krebs' solution (in mM: 117 NaCl, 3.6 KCl, 2.5 CaCl_2_, 1.2 MgCl_2_, 1.2 NaH_2_PO_4_, 25 NaHCO_3_, and 11 D-glucose) for a blind ruptured patch-clamp recording as our previous study [33]. Resistance of the patch electrodes was typically 4~10 MΩ. The internal eletrode solution contained (in mM: 135 K-gluconate, 0.5 CaCl_2_, 2 MgCl_2_, 5 KCl, 5 EGTA, 5 HEPES and 5 D-glucose). Currents were filtered at 2 kHz and digitized at 5 kHz (Axopatch 200B amplifier, Molecular Devices) and were analyzed by using pCLAMP8.5 program. The membrane potential was hold at -70 mV. To evoke Aδ- and C-fiber activation, the dorsal root stimulation was delivered with a suction electrode which was linked to a constant-current stimulator (Digitimer). Monosynaptic eEPSC was studied in the presence of 20 μM bicuculline and 2 μM strychnine. Frequency and amplitude of sEPSC were analyzed with Axograph (Molecular Devices). Afferent Aδ- or C-fibers were identified by the basis of the conduction velocity (CV) of afferent fibres (Aδ: 2~12 m/s; C: <1.2 m/s) calculated from the latency of EPSC from a stimulus artifact, the length of dorsal root, and the stimulus threshold (Aδ: 10~60 μA; C: 180~620 μA). The Aδ and C responses were considered as monosynaptic in origin when the latency remained constant and there was no failure during stimulation at 20 Hz for the Aδ-fiber evoked EPSCs, and at 2 Hz for the C-fiber evoked EPSCs. Drugs were applied through a superfusion exchange of the solutions in the recording chamber. The drugs used in the present studies included strychnine, bicuculline, MPP, 17β-estradiol (Sigma, USA), BIM and H89 (Calbiochem, USA). All drugs except for MPP, 17β-estradiol and H89 (where dimethyl sulphoxide was used as a solvent) were dissolved in distilled water at 1000 times the concentration in stock and kept at -20°C. On the experimental days, they were diluted to the desired concentration within Kreb's solution.

### Data analysis and statistics

All numerical data were presented as the mean ± S.E.M. Statistical significance was determined as P < 0.05 using the student's paired *t*-test, *n *refers to the number of neurons studied.

## Competing interests

The authors declare that they have no competing interests.

## Authors' contributions

XZ and KCL conceived and designed the study. YQZ performed the experiments. All authors read and approved the final manuscript.
